# Central Australian Aboriginal women’s pregnancy, labour and birth outcomes following maternal smokeless tobacco (pituri) use, cigarette use or no-tobacco use: a prospective cohort study

**DOI:** 10.1186/s12889-021-10872-z

**Published:** 2021-04-28

**Authors:** Angela Ratsch, Fiona Bogossian, Kathryn Steadman

**Affiliations:** 1 Wide Bay Hospital and Health Services, Hervey Bay, Queensland 4655 Australia; 2 Professor of Practice Education in Health at the University of the Sunshine Coast (USC) and USC Academic Lead at the Sunshine Coast Health Institute (SCHI), Birtinya, Queensland 4575 Australia; 3 Associate Professor School of Pharmacy, The University of Queensland, Brisbane, Queensland 4102 Australia

**Keywords:** Pregnancy, Maternal outcomes, Smoking, Smokeless tobacco, Pituri, Central Australia, Australian Aboriginal and Torres Strait Islander people

## Abstract

**Background:**

Outcomes related to maternal smoked tobacco (cigarette) use have been substantially examined over the past 50 years with resultant public health education targeted towards the reduction of use during pregnancy. However, worldwide the effects of maternal smokeless tobacco use have been less well explored and in Australia, there has been no examination of maternal outcomes in relation to the use of Australian *Nicotiana* spp. (tobacco plant) as a smokeless tobacco, colloquially known as pituri. The aim of this study is to describe the maternal outcomes of a group of central Australian Aboriginal women in relation to their self-reported tobacco use.

**Methods:**

Eligible participants were > 18 years of age, with a singleton pregnancy, > 28 weeks gestation, and who planned to birth at the Alice Springs Hospital (the major regional hospital for central Australia, in the Northern Territory, Australia). The sample consisted of 73 conveniently recruited women categorized by tobacco-use status as no-tobacco users (*n* = 31), pituri chewers (*n* = 19), and smokers (*n* = 23).

**Results:**

There were differences in the groups in relation to teenage pregnancies; 35% of no-tobacco users, compared with 5% of pituri users, and 13% of smokers were <  20 years of age. The chewers had a higher rate (48%) of combined pre-existing and pregnancy-related elevated glucose concentrations compared with smokers (22%) and no-tobacco users (16%).The pituri chewers had the lowest rate (14%) of clinically significant post-partum hemorrhage (> 1000 ml) compared with 22% of smokers and 36% of the no-tobacco users.

**Conclusions:**

This is the first research to examine pituri use in pregnancy and the findings indicate possible associations with a range of adverse maternal outcomes. The use of smokeless tobacco needs to be considered in maternal healthcare assessment to inform antenatal, intrapartum and postpartum care planning.

**Implications for public health:**

Female smokeless tobacco use is a global phenomenon and is particularly prevalent in low and middle income countries and in Indigenous populations. The findings contribute to the developing knowledge around maternal smokeless tobacco use and maternal outcomes. Maternal screening for a broader range of tobacco and nicotine products is required.

**Note to readers:**

In this research, the central Australian Aboriginal women chose the term ‘Aboriginal’ to refer to themselves, and ‘Indigenous’ to refer to the broader First Peoples. That choice has been maintained in the reporting of the research findings.

## Background

In 1957, Simpson [1] reported an association between maternal tobacco smoking and adverse maternal and perinatal outcomes. Those findings initiated a public health research agenda focused on the impact of smoking in pregnancy. Maternal tobacco smoking, and maternal exposure to combusted tobacco vapours are now recognized as leading modifiable risks associated with adverse maternal and perinatal outcomes [2, 3].

However, globally the use of smokeless tobacco (ST) exists alongside of, and in some localities, exceeds the use of smoked tobacco [4]. The collective term ST describes non-combusted tobacco products that are sucked, chewed, or applied to the gums or nasal lining as powders and pastes. Common ST products include dip, spit, chew, snus, nass and pituri. The World Health Organization (WHO) acknowledges that quantifying worldwide ST use is hampered by the absence of data from 58 member states (including Australia and New Zealand) and inadequate data collection by many of the reporting member states, with often a once-only report [4].

Nevertheless, the most recent information from the 70 countries where ST use has been recorded for more than two tobacco use surveys estimates 90 million women (> 15 years of age) and a further 4.3 million girls (13–15 years of age) use ST [4, 5]. In the Northern Territory (NT) of Australia, central Australian Aboriginal populations utilize wild tobacco plants (*Nicotiana* spp.) called *pituri*, as ST [6]. The plants are masticated and retained in the lip/buccal space for lengthy periods of time in a practice colloquially known as ‘chewing’. When not in the lip/buccal space, the wet quid of tobacco is often stored behind the ear; potentially enabling transdermal nicotine administration [7]. Pituri use continues throughout pregnancy and lactation [8].

There is scant global research on maternal ST exposure. The research that exists shows that maternal ST use is associated with adverse maternal and fetal outcomes [9–13]. There is no research examining the effects of pituri use on pregnancy outcomes.

## Methods

This paper is a component of a larger descriptive research study, the protocol of which is published elsewhere [14]. This aspect of the study sought to answer the following question, ‘*What are the demographic, pregnancy and labour characteristics of Aboriginal women who self-report no-tobacco use, chewing pituri or smoking during pregnancy?*’ Neonatal outcomes, and maternal and neonatal tobacco and nicotine biological concentrations and their relation to pregnancy outcomes are reported elsewhere. The research was informed and overseen by a regional Aboriginal Women’s Council who provided design and methodology direction and approval for the protocols including recruitment processes and participant questionnaires. Ethical approval was obtained from the Central Australian (#2010.06.04) and The University of Queensland Human Research Ethics Committees (#2010000548 and # 2015001429). All participants provided written informed consent prior to enrolling in the study and all methods were performed in accordance with the guidelines and regulations of the National Statement for the Ethical Conduct in Human Research [15].

### Sampling frame

There had been no research conducted on the use of pituri in pregnancy or its outcomes to inform the research measures and thus the sampling frame. The most recent information on general ST use reported a rate of 38% by Aboriginal women across the NT in 1986–1987 [16]. A later observation of pregnant central Australian Aboriginal women indicated approximately 33% of women smoked, 33% of women chewed pituri, and 33% did not smoke or use pituri (R. Carroll, personal communication, 16 July, 2009). This smoking rate is consistent with rates reported in the NT Mothers and Babies Reports from 2003 onwards. Those reports indicate a lower rate of smoking (30%) for Aboriginal mothers in the Alice Springs District compared with an NT wide rate of 52% [17], and it is suggested that this is a result of the ‘local practice of chewing tobacco (pituri) in [that] region’ [18, 19].

The sample population included no-tobacco users (as the control group), ST users and smokers. Inclusion of smokers enables comparison of clinical outcomes between the different tobacco exposure methods. No known predictors of adverse maternal outcomes were cause for exclusion to the study. This was due to the endemic incidence and prevalence in the study population of the social and physiological predictors including: poverty, geographic isolation, lower levels of education, poor and overcrowded housing, high levels of domestic violence, anemia, high levels of alcohol use, poor nutrition, hypertension, diabetes and tobacco use [19–21]. Exclusion based on these endemic risk factors would have precluded most of the potential population from the study. The only exclusion criterion was self-reported dual pituri and cigarette use. This was due to the inability of the maternal outcome variables to categorically discriminate between the maternal effects of inhaled combusted tobacco compared with tobacco administered through the oral and nasal mucosal routes [7].

The purpose of the research was to conduct the first descriptive study around pituri use and maternal and perinatal outcomes. The authors recognised early in the study design that there was no previous research to inform the sample size and in addition, detecting adverse maternal outcomes attributable to tobacco exposure as a single independent variable requires an extensive sample size which was not feasible due to the relatively small year-on-year central Australian Aboriginal birthing population. Based on the observed rates of pituri use, smoking and no-tobacco use, a sample of 20 participants in each group was considered sufficient to provide preliminary data to inform further studies.

### Population and recruitment

The population consisted of conveniently recruited self-identified central Australian Aboriginal women, > 18 years of age, with a singleton pregnancy, > 28 weeks gestation, and who planned to birth at the Alice Springs Hospital. Recruitment videos detailing the research and participation requirements were constructed in the central Australian Aboriginal languages of Arrernte, Pitjantjatjara, Warlpiri, and in English by senior central Australian Aboriginal women. The videos were played in the antenatal waiting area of the Alice Springs Hospital. Potential participants were identified by researchers when they attended for an antenatal appointment or care at the Alice Springs Hospital. Participation was not sought based on known tobacco use status and no incentive was offered for enrolment.

The researchers were Aboriginal Health Workers (AHWs), Aboriginal Liaison Officers (ALOs) and midwives specifically trained in the use of the research tools and skilled in communicating sensitively with Aboriginal women from central Australia. As part of the enrolment process, the appropriate recruitment video was replayed to individual potential participants if they indicated interest in the project.

### Data collection

AHWs, ALOs and midwives were involved in the data collection. The data collection comprised two data collection strategies related to this pregnancy. First, an interview consisting of 23 semi-structured demographic and tobacco-use questions was used to collect information not normally recorded elsewhere. The questions around tobacco smoking and chewing including age of commencement, current frequency, changes during pregnancy, and for chewing, how was their pituri prepared and who else in their family chews. The interview was conducted antenatally, and in private and during the enrolment process. Informed by the literature [12, 22–30], the second strategy was the extraction of pregnancy, labour and birth information from CARESYS® (the NT electronic medical record system) following birth.

### Data analysis

Data from the interview and extracted data from the CARESYS® were de-identified and imported into SPSS®. Interview data was used to categorize participants into tobacco use groups: (a) no-tobacco user, (b) chewer, or (c) smoker. Missing data and outliers are reported but excluded from analysis. Descriptive statistics including frequency and proportions for categorical data and medians with ranges, or means with standard deviations if normally distributed, for continuous data are reported. Comparative analyses were undertaken using self-reported tobacco use as the independent variable for differences between categorical variables. Logistic regression was used to assess the association between elevated maternal glucose levels and tobacco-use group. Confidence intervals are reported at the 0.05 alpha level. The variables were broadly grouped as follows for the analysis:

- Demographic (age, residential address, education level)
- Lifestyle factors (alcohol and tobacco use)
- Past and current medical history (cardiac, hypertension, diabetes and renal disease)
- Pregnancy related (parity, gravida, elevated glucose, hypertension, STI, UTI, anemia, number of antenatal visits). Any CARESYS® report of diabetes (pre-gestational or gestational) was categorized as “elevated glucose” and likewise any report of hypertension (pre-gestational or gestational) as “elevated blood pressure” for the analysis in this study.
- Labour and birthing (LUSCS and post-partum hemorrhage)
- Birth outcome (viability)

In order to establish external validity of the sample, their data was compared with local, NT and Australian Indigenous maternal data.

## Results

### Demographic characteristics

#### Comparative data

A total of 73 central Australian Aboriginal women took part in the study (no-tobacco users *n* = 31, pituri users *n* = 19, and smokers *n* = 23). Table 1 shows that less than one third (31.5%) of the participant cohort reported smoking tobacco in the first 20 weeks of pregnancy compared with rates of around 50% in both the NT and Australian Indigenous populations. The use of alcohol was however similar in the participant group (14%) and the broader Indigenous population group (16%). The sample had a mean age of 24.5 years, similar to the mean age of the NT and Australian Indigenous birthing populations (25 years and 25.3 years respectively). In Australia, teenage pregnancy is classified as < 20 years of age [35], and while there were similar proportions in each of the comparator groups in this category (sample 20%, NT Indigenous mothers 20%, and Australian Indigenous mothers 18%), in the sample, there were less mothers > 35 years (2.7%) compared with the wider Indigenous Australian population (10%).

**Table 1:** Maternal participant characteristics in comparison with Australian Indigenous birthing populations

Variable	Participants %	Comparative populations (where data available)^e^		
		Indigenous Alice Springs mothers^a^%	Indigenous NT mothers^a,b^%	Indigenous Australian mothers^a,b,c^%
Age at birthing				
Mean (years)	24.5	25.8	25.0	25.3
< 20	20.5	21.0	20.0	17.8
21–34	76.8	70.8	72.5	72.2
> 35	2.7	8.2	7.5	10.0
Usual residence^d^				
Urban	16.4	39.3	38.4	42.1
Regional	n/a	n/a	n/a	34.9
Rural/remote	83.5	60.6	61.6	23.0
Tobacco use (smoking only)				
First 20 weeks	31.5	35.0	52.0	47.0
Alcohol in pregnancy	14.2		9.5	16.3
Parity (*n* =)				
0	34.3		33.5	33.9
1–2	48.6		42.0	44.6
3+	17.1		24.4	23.3
Antenatal visits (*n* =)				
1–3	16.1	13.4	8.1	
4–7	27.4	35.9	30.5	(≥ 5) 85.0
8 and more	56.4	49.3	59.8	
Gestation at first antenatal visit				
< 14 weeks	51.5	61.25	61.0	52.5
14–26 weeks	34.4	29.5	31.6	(< 20 weeks) 74.9
> 27 weeks	14.0	9.2	7.4	(> 20 weeks) 25.1

^a^Hall, J., Case, A., & O’Neil, L. (2015). *Northern Territory Midwives’ Collection. Mothers and Babies 2013* [31]

^b^Australian Institute of Health and Welfare. (2015). *Australia’s Mothers and Babies 2013-in brief*. *Perinatal Statistics Series no. 31* [32]

^c^Australian Institute of Health and Welfare. (2013). *Healthy for Life: results for July 2007–June 2011*. (IHW 84). Canberra: AIHW [33]

^d^Remoteness area derived by applying ABS 2011 Australian Statistical Geography Standard (ASGS) [34] to area of mother’s usual residence. At the Australian level, no regional area is defined for the NT. NT Mothers and Babies Report [31] defines urban as the NT townships including Alice Springs; rural/remote covers the rest of the NT

^e^Percentages have been rounded for reporting purposes and may not add to 100%

Parity was comparable across the groups with 33–34% nulliparous, and approximately 44–49% with 1–2 previous births. The data shows that the number of participants with more than eight antenatal visits (56%) was comparable with the wider NT group (60%), although the number of participants with a low number of antenatal visits (1–3 visits) was 16%, twice that of the NT group (8%). Likewise, the proportion of the participant group who attended their first antenatal visit after 27 weeks gestation (14%) was double that of the NT group (7%).

#### Sample data

The sample consisted of participants from across the geographical research area (0–1020 km from Alice Springs) and at the time of birth, the median maternal age of the participant group was 24 years with no significant difference between groups (Table 2). There was a difference in the groups in relation to teenage pregnancies with 35% of no-tobacco users, compared with 5% of pituri users, and 13% of smokers being < 20 years of age. There was also a difference in the school leaving grade of participants; the chewers had a lower median leaving grade and the smokers had a higher median leaving grade. There was no difference between the groups based on alcohol use.

**Table 2:** Maternal demographics according to maternal self-reported tobacco use (*N* = 73)

Variable	Maternal self-reported tobacco use			
	Total *N* = 73	No-tobacco use *n* = 31	Chewer *n* = 19	Smoker *n* = 23
Maternal age in years				
Median (range)	24 (18–38)	22 (18–37)	26 (18–34)	23 (18–38)
Maternal age categorized, n (col %, 95 CI)				
< 20	15 (21, 13–32)	11 (35, 20–54)	1 (5,1–31)	3 (13, 4–35)
20–29	41 (56, 44–67)	15 (48, 31–66)	10 (53, 30–74)	16 (70, 48–85)
30–39	17 (23, 15–35)	5 (16, 7–34)	8 (42, 22–65)	4 (17, 6–39)
Median (range)	10 (5–12)	9.5 (5–12)	8.5 (5–11)	10 (7–12)
Left school in/at Grade, n (col %, 95 CI)				
≤ 7	13 (22, 13–34)	4 (17, 6–38)	7 (39, 19–63)	2 (11, 3–36)
8–12	47 (78, 66–87)	20 (83, 62–94)	11 (61, 37–81)	16 (89, 63–97)
Unknown^a^	13	7	1	5
Residential distance from Alice Springs - kilometres				
Median (range)	294 (0–1020)	195 (0–700)	350 (0–1020)	294 (0–788)
Urban or rural/remote^b^, n (col %, 95 CI)				
Urban	12 (16, 9–27)	6 (19, 9–38)	2 (11, 2–35)	4 (17, 6–39)
Rural/remote	61 (84, 73–91)	25 (81, 62–91)	17 (89, 65–98)	19 (83, 61–94)
Reported alcohol use, n (col %, 95 CI)				
Yes	10 (14, 8–24)	4 (13, 5–30)	3 (17, 5–42)	3 (13, 5–30)
No	62 (86, 76–92)	27 (87, 70–95)	15 (83, 58–95)	20 (87, 65–96)
Unknown^a^	1		1	

^a^Missing values (unknown) not included in %

^b^Urban or rural/remote dichotomized as residing < 20 km> from delivery hospital

### Previous pregnancy and birth outcomes

This was the first pregnancy for 12 no-tobacco users, three chewers, and four smokers which influences the differences in parity and previous livebirths (Table 3). One participant in each group had previously experienced a stillbirth, and a chewer and a smoker each previously experienced a neonatal death.

**Table 3:** Previous pregnancy(s) and outcomes according to maternal self-reported tobacco use (*N* = 73)

Variable	Maternal self-reported tobacco use			
	Total *N* = 73	No-tobacco use *n* = 31	Chewer *n* = 19	Smoker *n* = 23
	n (column %, 95 confidence interval)			
Gravida				
1	19 (27, 18–39)	12 (40, 24–59)	3 (17, 5–42)	4 (18, 7–41)
2–3	33 (47, 36–59)	13 (43, 27–62)	10 (56, 32–77)	10 (45, 26–67)
4+	18 (26,17–37)	5 (17, 7–35)	5 (28, 12–53)	8 (36, 19–59)
Unknown^a^	3	1	1	1
Parity				
0	24 (34, 24–46)	17 (57, 38–73)	3 (17, 5–42)	4 (18, 7–41)
1–2	34 (49, 37–60)	10 (33, 19–52)	12 (67, 42–85)	12 (55, 33–74)
3+	12 (17, 10–28)	3 (10, 3–28)	3 (17, 5–42)	6 (27, 12–50)
Unknown^a^	3	1	1	1
Birth outcomes				
Livebirths				
0	25 (34, 24–46)	16, (52, 34–69)	3 (16, 5–40)	6 (26, 12–48)
1–2	33 (45, 34–57)	11 (35, 20–54)	12 (63, 39–82)	10 (43, 25–64)
3+	15 (21, 13–32)	4 (13, 5–30)	4 (21, 8–46)	7 (30, 15–52)
Stillbirths	3	1	1	1
Neonatal deaths	2	0	1	1

^a^Unknown/missing data not included in %

### Current pregnancy: antenatal care and pregnancy complications

Table 4 shows that the median gestation at first ultrasound for all participants was 15 weeks, ranging from 6 to 37 weeks with no differences between the groups. Similarly, there was no difference in gestation at first antenatal visit (median 14, range 5–38), or the number of antenatal visits (median 8, range 1–20). The chewing group had a very low frequency of anemia (*n* = 1, 5%) and hypertension (*n* = 1, 5%) in comparison with the smokers (*n* = 6, 26% and *n* = 5, 22% respectively) and the no-tobacco users (*n* = 8, 26% and *n* = 7, 23% respectively).

**Table 4:** Current pregnancy characteristics according to maternal self-reported tobacco use (*N* = 73)

Variable	Maternal self-reported tobacco use			
	Total *N* = 73	No-tobacco use *n* = 31	Chewer *n* = 19	Smoker *n* = 23
	n (col %, 95 Confidence interval)			
Number of antenatal visits				
1–3	10 (16, 9–28)	4 (14, 5–33)	2 (13, 3–43)	4 (21, 8–46)
4–7	17 (27, 18–40)	8 (29, 15–48)	5 (33, 14–61)	4 (21, 8–46)
8 and more	35 (56, 44–68)	16 (57, 38–74)	8 (53, 28–77)	11 (58, 35–78)
Missing^a^	11	3	4	4
Median, (Range)	8, (1–20)	8.5, (1–18)	8, (2–20)	9, (2–16)
Gestation at 1st antenatal visit in weeks				
< 14 weeks	32 (50, 38–62)	15 (58, 38–75)	5 (29, 12–56)	12 (57, 35–77)
14–26 weeks	23 (36, 25–49)	7 (27, 13–48)	11 (65, 39–84)	5 (24, 10–47)
> 27 weeks	9 (14, 7–25)	4 (15, 6–36)	1 (6, 1–34)	4 (19, 7–42)
Missing^a^	9	5	2	2
Median, (Range)	14, (5–38)	12.5, (5–35)	18, (7–31)	12, (5–38)
Gestation at 1st ultrasound in weeks				
< 14	31 (46, 34–59)	15 (54, 35–71)	5 (29, 12–55)	11 (50, 29–71)
14–26	27 (40, 29–53)	11 (39, 23–59)	10 (59, 34–80)	6 (27, 12–50)
> 27	9 (13, 7–24)	2 (7, 2–25)	2 (12, 3–39)	5 (23, 9–45)
Missing^a^	6	3	2	1
Range	15, (6–37)	12.5, (6–31)	16, (7–30)	14.5, (6–37)
Elevated glucose^b^				
Yes	19 (26, 17–38)	5 (16, 7–34)	9 (47, 26–70)	5 (22, 9–44)
No	54 (74, 62–83)	26 (84, 66–93)	10 (53, 30–74)	18 (78, 56–91)
Odds ratio (95%CI) of elevated glucose		Reference group	4.68 (1.26–17.42)	1.44 (0.36–5.73)
Hypertension^b^				
Yes	13 (18, 10–29)	7 (23, 11–41)	1 (5, 1–31)	5 (22, 9–44)
No	60 (82, 71–90)	24 (77, 59–89)	18 (95, 69–99)	18 (78, 56–91)
Anemia				
Yes	15 (21, 13–32)	8 (26, 13–44)	1 (5, 1–31)	6 (26, 12–48)
No	58 (79, 68–87)	23 (74, 56–87)	18 (95, 69–99)	17 (74, 52–88)
Sexually transmitted infection				
Yes	14 (19, 12–30)	6 (19, 9–38)	6 (32, 14–56)	2 (9, 2–30)
No	59 (81, 70–88)	25 (81, 62–91)	13 (68, 44–86)	21 (91, 70–98)
Urinary tract infection				
Yes	8 (11, 5–21)	3 (10, 3–26)	3 (16, 5–40)	2 (9, 2–30)
No	65 (89, 79–95)	28 (90, 73–97)	16 (84, 60–85)	21 (91, 70–98)
Renal disease				
Yes	3 (4, 1–12)	3 (10, 3–27)	0	0
No	70 (96, 88–99)	28 (90, 73–97)	19 (100, −)	23(100, −)
Cardiac disease				
Yes	7 (10, 5–19)	4 (13, 5–30)	1 (5, 1–31)	2 (9, 2–30)
No	66 (90, 81–95)	27 (87, 70–95)	18 (95, 69–99)	21 (91, 70–98)

^a^Missing data not included in percentage

^b^CARESYS® data not specific to identify if condition was pre-existing or gestational

Maternal elevated glucose was more evident in the chewing group (*n* = 9, 47%) compared with the smoking group (*n* = 5, 22%) and the no-tobacco group (*n* = 5, 16%) (*p* = 0.04). Further examination of this association revealed that the ST group were more than four times as likely to have raised glucose levels compared to no-tobacco-users (OR: 4.68, 95% CI 1.26–17.42). Compared to no-tobacco users, smokers were more likely to have raised glucose levels, but the difference was not statistically significant (OR: 1.44, 95% CI 0.36–5.73).

### Current pregnancy: place of labour, outcomes and complications

One chewer and one smoker birthed prior to arrival at hospital, and one chewer and one smoker birthed in Port Augusta and Katherine respectively (Table 5). Two mothers (one no-tobacco use and one smoker) were transferred to Adelaide prior to labour due to fetal congenital abnormalities. There were two stillbirths (both at 40 weeks gestation) in the cohort, one no-tobacco user and one chewer. The proportion of chewers (21%) who experienced Lower Uterine Segment Cesarean Section (LUSCS) was less than half the rate of the smoking (48%) and no-tobacco group (39%).

**Table 5:** Place of birth, birth and labour outcomes according to maternal self-reported tobacco use (*N* = 73)

	Maternal self-reported tobacco use			
	Total *N* = 73	No-tobacco use *n* = 31	Chewer *n* = 19	Smoker *n* = 23
	n (column %, 95 confidence interval)^b^			
Birth location				
Alice Springs Hospital	67 (92, 83–96)	30 (97, 79–99)	17 (89, 65–98)	20 (87, 65–96)
Other hospital^a^	4 (3, 1–11)	1 (3, 1–21)	1 (5, 1–31)	2 (9, 2–30)
Born before arrival (BBA)	2 (5, 2–14)	0	1 (5, 1–31)	1 (4, 1–27)
Method of birthing				
Spontaneous vaginal delivery	43 (59, 47–70)	18 (58, 40–74)	14 (74, 49–89)	11 (48, 28–68)
Forceps	2 (3, 1–11)	1 (3, 1–21)	0	1 (4, 1–27)
Ventouse	1 (1, 1–10)	0	1 (5, 1–31)	0
Elective LUSCS	10 (14, 7–24)	2 (6, 2–23)	3 (16, 5–40)	5 (22, 9–44)
Emergency LUSCS	17 (23, 15–35)	10 (32, 18–51)	1 (5, 1–31)	6 (26, 12–48)
Post-partum hemorrhage^c^	30 (41, 100)	14 (45, 47)	7 (37, 23)	9 (39, 30)
Blood loss mL, Median (range)	675 (500–1670)	900 (500–1670)	650 (500–1300)	550 (500–1100)
Blood loss 500–999 mL	22 (30, 21–42)	9 (29, 16–48)	6 (32, 14–56)	7 (30, 15–52)
Blood loss > 1000 mL	8 (11, 5–21)	5 (16, 7–34)	1 (5, 1–31)	2 (9, 2–30)
Viability				
Liveborn	71 (97, 89–99)	30 (97, 79–99)	18 (95, 69–99)	23 (100, −)
Still born	2 (3, 1–11)	1 (3, 1–21)	1 (5, 1–31)	0 (−)

^a^Other hospital Katherine, Port Augusta and Adelaide hospital

^b^Percentages have been rounded for reporting purposes and may not add to 100%

^c^Post-partum hemorrhage is quantified as > 500 ml within the first 24 h of birth

There were no reports of antepartum hemorrhage. Forty-one percent of the participants had a post-partum hemorrhage (greater than 500 mL blood loss). The lowest rate of post-partum hemorrhage (PPH) was in the chewing group (37%) and the highest rate was in no-tobacco group (45%). The no-tobacco group had the highest median PPH blood loss (900 mL) compared with the lowest median loss in the smokers (550 mL). Group differences were also noted in the percentage of clinically significant blood losses (> 1000 mL) with 36% of the no-tobacco users who hemorrhaged losing more than 1000 mL (16% of total group) compared to 22% of smokers (9% of total group) and 14% of chewers (5% of total group). Across the sample, one retained placenta was recorded to a chewing mother.

## Discussion

Smoking in pregnancy continues to be a phenomenon despite more than 50 years of evidence related to adverse maternal and perinatal outcomes and sustained public health campaigns disseminating this evidence. While the self-reported rate of smoking in pregnancy in the Australian Aboriginal population is 44% [35], the use of ST is not reported in this population, nor in the wider Australian maternal population. This study is a component of the larger research study and sought to address the question, *What are the demographic, pregnancy and labour characteristics of Aboriginal women who self-report no-tobacco use, chewing pituri or smoking during pregnancy?*’ The findings show that approximately 58% of the cohort reported tobacco use, comprising of 26% ST use and 32% cigarette use. Some differences between the tobacco-use groups were noted, including school leaving grade, age and parity. The most clinically significant differences were as follows:

### Hypertension

In the study cohort, one chewing participant (5%) was recorded as having elevated blood pressure compared with *n* = 5 (22%) of the smokers and *n* = 7 (23%) of the no-tobacco users; these findings may be confounded by the presence of cardiac and renal disease in those groups. Nicotine is a potent vasoconstrictor [36], however, smoking in pregnancy demonstrates a counter-intuitive, paradoxical, dose-dependent, inverse relationship between exposure and the development of pregnancy-related hypertension (in all forms) [37, 38] with on-going research to determine the mechanism [27, 39]. Whilst a slightly reduced adjusted OR for hypertension in pregnancy was reported in Alaskan smokeless tobacco users compared to non-ST users [12], other ST literature demonstrates reversed findings with some types of tobacco preparations increasing the risk of hypertension in pregnancy, for example, mishri (tobacco containing tooth cleaning powder) [40] and commercial Swedish snuff [41, 42].

### Anemia

In parallel with the finding of a reduced rate of hypertension in the chewing cohort, the research found only one chewer (5%) with maternal anemia compared with an equi-prevalence of 26% in the no-tobacco users (*n* = 8) and smokers (*n* = 6). In the literature, smoking during pregnancy is linked with maternal and fetal anemia [43]. ST literature reports either no effect or lower hemoglobin levels with mishri use [11, 40, 44]. Nevertheless, there may be endemic confounders in the participant sample that impact the presence of anemia that were not measured in this research, specifically the presence of intestinal parasites. Parasitic infections produce a range of nutritional disturbances including iron deficiency anemia [45]. In the NT, *Hymenolepis nana* is endemic and approximately 18% of infections are associated with anemia [46]. Interestingly, traditional knowledge dating from at least 1577 [47, 48] documents tobacco concoctions as an anthelmintic treatment. More recent evidence supports the hypothesis that higher nicotine exposure is associated with decreased intestinal worm burden [49]. It may be that the swallowing of small amounts of tobacco juices from pituri use impacts on intestinal parasite load, resulting in a reduction in anemia from that cause.

### Elevated glucose

In pregnancy, the development of insulin resistance is a normal maternal physiological change [50], nonetheless, cigarette smoking pre-gravida significantly increases the risk of gestational diabetes [51, 52]. Those findings were supported in this research with approximately 22% of smokers and almost half of the ST group having elevated glucose levels compared to 16% of no-tobacco users.

A longitudinal study involving over 114,000 females examined the relationship between female cigarette use and diabetes, and demonstrated a statistically significant dose-dependent adjusted risk of 1.42 for the development of diabetes in smokers [53]. Further research showed that for women, passive, past and active smoking increases the statistically significant RR of diabetes (occasional passive exposure 1.10; regular passive exposure 1.16; past smokers 20–29 years post cessation 1.17; current smokers 1–14 cigarettes a day 1.39; ≥ 25 cigarettes a day 1.98) [54]. Although not all studies demonstrate this risk [55], several large research activities [56–58] confirm an increased risk of impaired glucose regulation involving hyperinsulinemia and insulin resistance in smokers, increasing the risk of type 2 diabetes independent of a range of factors.

Research examining this glucose/smoking association report ‘tobacco use’, which incorporates both smoking and ST [59–62], conflating tobacco and nicotine exposure with the other products of combustion in cigarette use. The few research publications focused upon ST usage and diabetes demonstrate higher triglycerides in the general population of users [63] and a dose-response increasing OR for the development of diabetes [64–66]. The comparative results between smoking and ST use are suggestive of a nicotine specific mechanism involved in the altered glucose metabolism as discrete from a mechanism that involves the elements of combusted tobacco.

### LUSCS

Smokers and no-tobacco users experienced over twice the percentage of LUSCS (43%) compared with the chewers (21%), and this between-group difference was not explained by the collected variables. The literature [36] shows that nicotine has a biphasic effect at nicotine acetylcholine receptors, with exposure increasing a range of maternal physiological responses, and subsequently fetal responses through the maternal-fetal transfer of nicotine [67]. Perhaps other compounds within smoked tobacco pharmacokinetics and metabolism contribute to the difference between smokers and chewers in relation to emergency LUSCS. In the literature, higher LUSCS rates are noted in the presence of elevated glucose [68]. In this research, the reverse was found with the chewers having the highest rate of elevated glucose and the lowest rate of LUSCS. Literature demonstrates higher LUSCS rates in the presence of hypertension and meconium staining [69], however these factors did not contribute to the LUSCS rate in chewers or smokers in this study.

### PPH

The rate for all PPHs was 41%, which far exceeds the Australian incidence rates of between 3 and 26% [70], the wide range due to inconsistent State and Territory definitions and collection practices. The global incidence rate of PPH > 1000 mL is estimated at 10.5% of live births, which matches our findings of 11%, whereas for Australia and other developed countries the rate is between 1 and 5% of live births [71]. In this study, chewers had the lowest rate of PPH (37%), followed by the smokers (39%) and the highest rate was in the no-tobacco users (45%). The no-tobacco users also experienced the largest median blood loss. There is strong evidence that smoking and nicotine exposure in vivo and in vitro impacts placental vascularisation and subsequently maternal and fetal hemodynamics [72–74]. Literature on the association between PPH and smoking is scant, however a PPH risk reduction (OR 0.56, *p* = 0.026, 95%CI [0.33–0.93]) was measured for current smokers in a United Kingdom population (*n* = 10,213) [75]. Maternal ST use is demonstrated to produce placental morphology changes, including increased stromal fibrosis and excessive subtrophoblastic basement thickening, thereby increasing the placental barrier and in turn reducing maternal/fetal exchange [76], but how this translates to the incidence of PPH is not clear at this time.

## Limitations

As with all observational studies, the method had several limitations. The quantification of adverse maternal outcomes associated with a single specific exposure requires a very large sample size which was not feasible in the limited central Australian Aboriginal birthing population. In this small descriptive study, the rarity of adverse maternal outcomes translates to low frequency of events which may underestimate or overestimate the effects and limits generalizability to the wider populations of pregnant ST users. In addition, in this research, maternal self-report was used as the validation of tobacco use which is consistent with the national and international framework for the reporting of cigarette use in pregnancy. Whilst being the standard antenatal global assessment method, England and Zhang [39] point out that this method contests reliability and validity, and therefore result interpretation. Their contention is supported by data from differing populations, with under-reporting in Scotland by 25% of pregnant women [77] and in the USA by 24–28% of pregnant women [78], although other authors demonstrate that across different population groups, self-reporting is a valid maker of exposure [79–82]. In addition, the frequency, dose and volume of smoked tobacco and chewed tobacco varied with each participant over the duration of their pregnancy, as did the tobacco and nicotine concentration of the cigarettes and *Nicotiana* spp. (and consequently the pituri quids) due to species, environment and preparation [83]. Furthermore, participants were not asked about environmental or secondary exposure to cigarettes.

Participants were recruited in a convenience manner when they presented for antenatal/birthing care after 28 weeks gestation. Consequently, potential participants who experienced early- and mid-pregnancy loss and birth or who were transferred to a tertiary facility prior to 28 weeks were not in the sampling frame, so the impact of tobacco and nicotine exposure on whole of pregnancy outcomes may be underestimated. Not all potential participants were approached and these losses to recruitment applied to the three groups. At times there was no trained researcher or interpreter available, or an approach was not considered to be in the best interest of the potential participant. Potential participants were not approached if community members were present in order to maintain privacy and to avoid “shaming” either the potential participant or community members. Blagg [84] points out that for Aboriginal people, “shame” is a complex, cultural paradigm which is “bound up with anxieties about a loss of social status, a feared mortification of a public self”. Anxiety and uncertainly is created if a person is favourably or unfavourably singled out and Aboriginal people will strive to avoid being “shamed”.

The sample collection methods were designed to be non-intrusive to the mother and have minimal impact on clinical staff, with clinical priorities taking precedence over data collection. Information from CARESYS® and the maternal interviews may have been incomplete or inaccurate, however missing information occurred in all three groups and no patterns of missing data were observed. The data collection included estimates to measure some variables for example, estimating PPH. Errors in measurement could have been made as interrater reliability was not established, however these estimates [67] are standard clinical practices, and any errors would likely have translated equally across the three groups.

## Conclusion, recommendations and implications for public health

The exposure to combusted tobacco in pregnancy has long been recognized as a contributing factor in adverse maternal outcomes [1]. In Australia and New Zealand, maternal tobacco-use research and the translation of findings to practice is almost exclusively framed within this Euro-centric tobacco exposure context. Accordingly, public health policies and public health education is targeted around reducing cigarette use and the exposure of populations to tobacco smoke. Currently in Australia, the sole assessment of maternal tobacco and nicotine exposure is maternal cigarette use which overlooks the use of non-combusted tobacco and nicotine by other means including chewing tobacco, e-cigarettes, and nicotine patches, gums and mists. Prior to this research, the maternal outcomes from the central Australian Aboriginal ST use population would have been incorporated into those of the no-tobacco use population, possibly underestimating the true contribution of tobacco and nicotine exposure in maternal outcomes. Further research around the use of pituri by maternal populations is required to extend the findings of this study.

Given the use of ST and the increase in novel nicotine and tobacco products alongside the use of cigarettes, it is timely to review the maternal assessment tools and modify the term “cigarette use” to “tobacco and nicotine use”. As a public health measure, this will improve the estimation of tobacco and nicotine use, enabling a more comprehensive and accurate determination of the effects of that exposure.

In Australia, central Australian Aboriginal populations have a pre-colonisation history of tobacco use [6], yet this exposure has not been considered in terms of pregnancy, labour and birth outcomes. This research provides the first evidence that maternal pituri use may be associated with adverse maternal outcomes, with the most important being an increased rate of elevated glucose. There is biological plausibility linking high nicotine exposure and the development of diabetes [57, 85]. For the mother, the long-term effect of gestational diabetes can result in a number of chronic health conditions including diabetes, cardiac and peripheral vascular disease and diabetic renal disease [57, 58, 65, 85–91], all of which have a high incidence rate in this population [92]. The higher prevalence of elevated glucose in the chewing cohort is complex and requires substantial multidisciplinary research to determine if a relationship exists between the use of pituri and the development of elevated glucose in its users. Given that diabetes and sequelae are endemic in central Australian Aboriginal populations, this research should be prioritized.

## Data Availability

The data generated or analysed during this aspect of the study is included in this published article.

## Abbreviations

AHWs: Aboriginal Health Workers
ALOs: Aboriginal Liaison Officers
CI: Confidence interval
LUSCS: Lower Uterine Segment Cesarean Section
ml: Millilitres
NT: Northern Territory
OR: Odds ratio
PPH: Post-partum hemorrhage
RR: Relative ratio
SD: Standard deviation
ST: Smokeless tobacco
WHO: World Health Organization
